# Mr. Hebb's Case of Small-Pox

**Published:** 1801-06

**Authors:** C. H. Hebb

**Affiliations:** Worcester


### *To the Editors of the Medical and Phyfical Journal.*

GENTLEMEN,

IF the following fact, from its infrequency, or from any trifling additional light it may throw upon the theory of variolous contagion, may be deemed worthy a place in your valuable Journal, it is entirely at your difpofal.

---

About mid-day on Tuefday, the 7th ult. Elizabeth Berry, of this city, being at her full time, was feized with pain in the head and back, great thirft and heat, which by the good women where fhe refided, were fuppofed to be the harbingers of approaching labour. On the following day they informed me of thefe circumftances, and alfo that fhe had never had the fmall-pox, and that it had lately been at the next door. When I confidered that the heat and thirft were not confequent upon, but coeval with the pain in the back, I ftrongly fufpected that it would turn out the eruptive fever of that difeafe. I intimated my fufpicions to the attending women, and begged they would carefully watch the appearance of any eruption. In the night I was called up to her; and about five o'clock in the morning, (April 9th) fhe was delivered of a boy. I called upon her in the courfe of the day; but although fhe continued very hot, no eruption had appeared: towards the evening, however, her nurfe obferved about half a dozen pimples upon her face, and the next morning, when I faw her, many had appeared, which gradually thickened and became confluent. On the Monday morning following, (being only four complete days after labour), I was defired to look at the child, as he had been for fome hours apparently unwell, and had puked confiderably. I found him hotter than ufual, and he moaned much. On the Wednefday morning, being fix days from his birth, I perceived a confiderable variolous eruption, which gradually increafed; and on the Tuefday following (April 21ft) put a period to his exiftence. The child fucked on the day after his birth. The mother, I am happy to fay, recovered.

It may not here be improper to obferve, that I have fixed the dates of the fymptoms and appearances as they came under my own eye; although, probably, they took place feveral hours previous, as the women fay they thought the child was unwell on the Sunday evening, and on the Tuefday evening they faw a few little fpots, which they took for the red-gum, but which, from the number of variolous eruptions I faw on the following morning, there is good reafon to believe were the fmall-pox.

Did

Did this child receive the infection before or after birth?

It has been generally admitted that the casual small-pox rarely produces its constitutional symptoms till ten or twelve days after infection; and Dr. Haygarth, who has paid the most minute and unremitting attention to every thing connected with variolous contagion, seems to place eight days as the shortest period that elapses between infection and eruptive fever. Most authors are also of opinion, that the small-pox is not contagious during the eruption. As this child, from my own observation, had decided marks of eruptive fever, when only four days old, and eruptions when only six, and these events (as before observed) had taken place several hours previous, it appears clear, if he received the infection after birth, that the above-mentioned positions cannot, in all cases, be well founded: but when I consider the high and numerous authorities that support these doctrines, my mind seems satisfied that he took the disease before that period. It required no additional facts to prove, that the foetus could receive the variolous infection in utero; there are so many on record, that the hardiest sceptic can no longer foster a doubt upon the subject, but I have not been able to discover one that could throw much light on the stage at which the mother conveyed it to the foetus, whether before the eruptive fever, during the eruption, or after it. If Dr. Haygarth's statements are founded in truth, this fact will at least prove that it may be conveyed even previous to the eruptive fever, as that fever took place in the child only six days from its first appearance in the mother, and eight days are allowed by Dr. H. and other able writers, to be the shortest period that passes between infection and constitutional symptoms.

Two facts related by Dr. Waterhouse, go far to prove that the disease may be communicated through the medium of the blood, sooner than in any other way. He instances two ladies, who took the small-pox from the effluvia of the blood of another lady, who was bled during the eruptive fever, it not being known at the time that she had that disease; the one lady held the bason, the other had it handed to her to put down. When we consider that the best anatomists are of opinion that the chief use of the placenta is as a breathing organ for the foetus, there seems a material analogy between these parts which mutually support and illustrate each other. If breathing for a few moments the effluvia of the blood, during the eruptive fever, can convey the small-pox, it requires no great stretch of faith to believe, that by that continual respiration (if I may use the expression) which is going on between the mother and foetus, it may be conveyed before that period.

I am, &c.

